# Molecularly Imprinted Polymer-Based Sensors for SARS-CoV-2: Where Are We Now?

**DOI:** 10.3390/biomimetics7020058

**Published:** 2022-05-06

**Authors:** Aysu Yarman, Sevinc Kurbanoglu

**Affiliations:** 1 Molecular Biotechnology, Faculty of Science, Turkish-German University, Sahinkaya Cad. 86, Beykoz, Istanbul 34820, Turkey; 2 Institute of Biochemistry and Biology, University of Potsdam, Karl-Liebknecht-Strasse 24-25, 14476 Potsdam, Germany; 3 Faculty of Pharmacy, Department of Analytical Chemistry, Ankara University, Yenimahalle, Ankara 06560, Turkey; skurbanoglu@ankara.edu.tr

**Keywords:** molecularly imprinted polymers, biomimetic sensors, SARS-CoV-2

## Abstract

Since the first reported case of COVID-19 in 2019 in China and the official declaration from the World Health Organization in March 2021 as a pandemic, fast and accurate diagnosis of severe acute respiratory syndrome coronavirus 2 (SARS-CoV-2) has played a major role worldwide. For this reason, various methods have been developed, comprising reverse transcriptase-polymerase chain reaction (RT-PCR), immunoassays, clustered regularly interspaced short palindromic repeats (CRISPR), reverse transcription loop-mediated isothermal amplification (RT-LAMP), and bio(mimetic)sensors. Among the developed methods, RT-PCR is so far the gold standard. Herein, we give an overview of the MIP-based sensors utilized since the beginning of the pandemic.

## 1. Introduction

**Figure 1:**
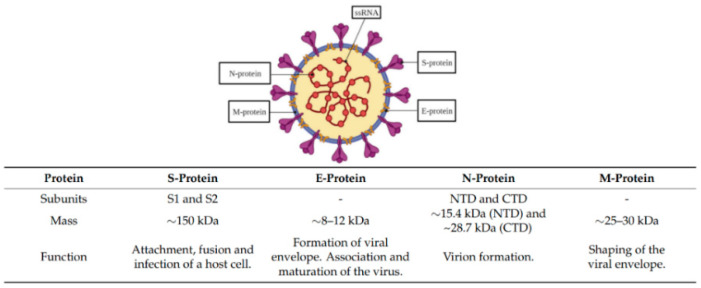
Schematic representation of structural proteins of severe acute respiratory syndrome coronavirus 2 (SARS-CoV-2) and properties Reprinted with permission from Ref. [5]. 2021, MDPI.

The outbreak of COVID-19 was first reported in December in Wuhan, China, and has spread worldwide. In March 2020, the World Health Organization (WHO) officially declared it a pandemic. The WHO reported that, since then, there have been globally 490.853.129 confirmed cases and 6.155.344 deaths (5 April 2022). The spread of the virus, SARS-CoV-2, is fast and can lead to symptoms such as fever, cough, and shortness of breath, while in some cases, no symptoms are exhibited [1,2]. It is a single-stranded RNA-enveloped virus, which belongs to the β coronavirus family [3]. The majority of immunoassays address the spike protein (S protein) or the nucleocapsid protein (N protein) (Figure 1). While the S protein binds to the host cell receptor angiotensin-converting enzyme 2 (ACE2) and mediates viral cell entry, the main role of the N protein is viral genome replication and transcription [3,4]. 

Different diagnosis strategies have been developed such as RT-PCR, immunoassays (ELISA: Enzyme-Linked Immunosorbent Assay, lateral flow immunoassay), CRISPR, lateral flow-based nucleic acid detection, RT-LA, bio(mimetic)sensors, and microarray-based analysis [1,2,5,6,7,8,9,10,11,12,13,14,15,16,17]. Among the described methods, RT-PCR is the gold standard.

Over the past decades, increasing attention has been given to the substitution of biological reagents in bioanalysis, separation techniques, and biotechnology by biomimetic materials such as fully synthetic organic polymers (molecularly imprinted polymers, MIPs) and aptamers [18,19,20]. IUPAC defines the term biomimetic as “Refers a laboratory procedure designed to imitate a natural chemical process. Also refers to a compound that mimics a biological material in structure or function“. The lotus effect at a water-repelling surface is the best-known example of biomimetic systems. One important motivation for the development and application of biomimetic recognition elements is their potentially higher stability and lower price as compared with biomolecules. Herein, we focus on only MIP-based biomimetic sensors and their potential for SARS-CoV-2 sensing. 

## 2. Molecularly Imprinted Polymers

The concept of molecular imprinting dates back to 1931 when Polyakov demonstrated specific adsorption properties of silica gel that recognized its target methyl orange [21]. Nevertheless, the synthesis of molecularly imprinted polymers (MIPs) was boosted by Wulff and Mosbach later in the 1970s [22,23]. Since then, a broad spectrum of analytes including low-molecular-weight molecules, such as pharmaceuticals, sugars, toxins, narcotic drugs, pesticides, and biomacromolecules such as proteins, nucleic acids, bacteria, and viruses have been described [20,24,25,26,27,28,29,30,31,32,33,34,35,36,37,38,39,40,41,42,43,44,45,46,47,48,49].

**Figure 2:**
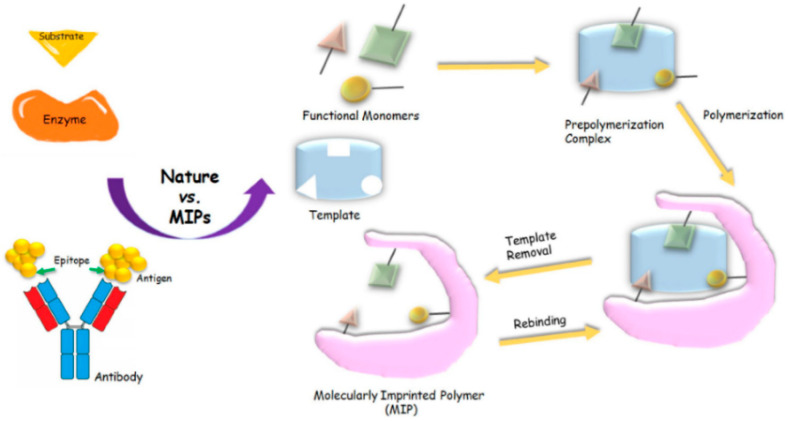
Workflow of MIP preparation Reprinted with permission from [20]. 2021, Elsevier.

Briefly, MIPs are prepared by copolymerizing functional monomers, cross-linkers (in the case of electropolymerization, there is no need to use cross-linkers), and the target analyte, the so-called template (Figure 2). Subsequent removal of the template leads to the formation of molecular cavities with a molecular memory, mirroring size, shape, and/or the functionality of the template. As the binding sites of MIPs mimics antibodies, they are also called artificial or tailor-made antibodies. The evolution of highly specific antibodies and efficient enzymes exploited the arsenal of 20 natural amino acids. Interestingly, the affinity of the MIPs, which are formed with only one functional monomer, reaches the values of natural antibodies [20]. Molecular modeling and the application of more than one functional monomer have the potential of further optimization. On the other hand, catalytically active MIPs that approach the activities of enzymes typically require the integration of analogs of prosthetic groups. MIPs are more stable under harsh conditions, such as extreme pHs, organic solvents, and high temperatures as compared to their biological counterparts [19,20,50]. 

### 2.1. Structural Levels Target Analytes

**Figure 3:**
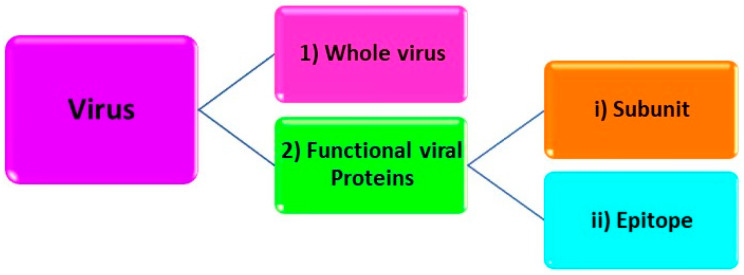
Schematic representation of structural levels of target analyte in the virus imprinting process.

As biomacromolecules and viruses have complex, flexible, and fragile structures, in addition to milder preparation conditions, alternative techniques comprising different structural levels of the target analyte have been utilized. Yarman and Scheller recently summarized these levels exemplifying proteins [19,51,52,53]. Similar approaches have also been applied to viruses (Figure 3). These approaches include:

(1) Whole virus imprinting: In this approach, a whole virus is used as a template [54,55,56,57]. In contrast to low-molecular-weight analytes, there are some obstacles when imprinting viruses. For imprinting, a high amount of pure virus is needed. Moreover, virus sample preparation requires appropriate laboratory, equipment, and experienced personnel [39]. Moreover, due to their large number of potential interaction sites and functional groups on their surfaces, heterogeneous binding sites and higher cross-reactivities can be obtained [20,37,39,55,58,59,60].

(2) Functional viral protein imprinting: Viruses consist of various proteins, which have different functions. Utilizing glycoprotein gp51 of bovine leukemia virus, the S or N protein of SARS-CoV-2 as templates for MIP preparation can be examples of this approach [61,62,63]. In addition to whole protein, subunits or peptide fragments have been applied as templates. 

(i) Subunit imprinting: Subunit imprinting is based on using the fragments of the viral protein as a template. Denizli’s group utilized the antigen-binding fragment (Fab) as a template for the determination of immunoglobulin G (IgG) on a surface plasmon resonance (SPR) chip. The MIP sensor could recognize both the target, Fab, and the whole IgG MIP synthesis on a SPR-chip. This Fab-imprinted polymer layer binds both the Fab fragment and the whole IgG molecule. Scheller’s group further extended this approach to oxidase (BMO) and reductase domains (BMR), which could recognize their targets or holo Cytochrome P450 BM3. This aspect has also been presented for SARS-CoV-2, in which the receptor-binding domain was used as a template [64,65].

(ii) Epitope Imprinting: To overcome the limitations in biomacromolecule and virus imprinting, exposed peptides of the analyte have been used as templates, which could recognize both the template and its holoprotein/whole virus [20,66,67,68,69,70,71,72,73,74,75]. This concept was introduced by Rachkov and Minoura and was termed epitope imprinting, as it is similar to the immunological determinant recognized by an antibody [20,76,77]. The first “epitope-MIP” was constructed via bulk imprinting, whereas Shea’s group immobilized the target on the support that was removed following the polymer formation generating the complementary cavities [20,78]. Later, Scheller’s group developed a fully electrochemical approach (including template removal: anodic potential pulses) on gold surfaces [79]. Epitope imprinting was also exploited for virus sensing for various viruses, comprising recently SARS-CoV-2 [39,58,72,80,81,82,83]. It is important to note that in the epitope approach, the area of specific interaction of the protein with the MIP is restricted to the epitope cavities. “Out-of-pocket interaction” of the protein/virus with the polymer surface can cause pronounced nonspecific binding [84]. On the other hand, interaction with the underlying support, e.g., metal electrodes, has to be taken into consideration for whole protein/virus MIPs [46]. 

### 2.2. Steps of MIP Preparation

In general, MIPs are prepared in three steps as described in Figure 2: 

(1) Formation of the pre-polymerization complex: In the first step, functional monomer(s) and the template molecules interact with each other to form the pre-polymerization complex. Two main approaches, namely covalent and noncovalent, have been described for the preparation of the pre-polymerization complex.

(i) Covalent approach: The covalent approach, which was introduced by Wulff and Sarhan [22], and Shea [85], is based on the formation of reversible covalent bonds between the template molecules and the functional monomer(s), followed by crosslinking. To remove the template from the polymer matrix, these chemical bonds must be cleaved, and rebinding occurs via the same covalent bonds [86,87]. This method results in the formation of the stable and stoichiometric pre-polymerization complex. Moreover, in contrast to the non-covalent approach more homogenous binding sites can be obtained. Nevertheless, this approach has some obstacles, especially a narrow template spectrum and slower binding kinetics as compared to the noncovalent approach.

(ii) Noncovalent approach: The noncovalent approach was developed by Arshady and Mosbach [23]. By contrast, in this approach, a pre-polymerization complex is formed via the noncovalent interactions, such as hydrogen bonds, ionic bonds, van der Waals forces, and hydrophobic interactions, between the template and the functional monomer(s) [87]. As it resembles molecular recognition in nature, it is also called the biochemists’ approach. Template molecules can be removed by simple solvent extraction, and rebinding of the analyte is again obtained by the same noncovalent interactions. Furthermore, the template spectrum is broad. However, the yield of binding sites is low compared to the covalent approach. 

To overcome the drawbacks of both approaches described above, the semi-covalent approach was developed by Whitcombe et al., which is a hybrid of two approaches [88]. In this approach, a pre-polymerization complex is formed via a covalent bond, and rebinding of the analyte is achieved by noncovalent interactions between the polymer and the analyte [87].

(2) Polymerization: The second step of MIP preparation is polymerization. Bulk polymerization is most frequently exploited for the preparation of MIPs among the different formats. With this technique, monolithic structures are produced, which must be then grounded and sieved. The disadvantages of this method are that it is time-consuming, and slow binding kinetics are obtained. To overcome these drawbacks, different methods have been introduced including suspension, emulsion, or precipitation polymerization, which result in the formation of micro- or nanobeads; MIP nanomaterials such as nanoparticles and nanospheres; MIP nanomaterial composites; self-assembled monolayers of thiols; the spreader-bar technique; stamping; and electropolymerization [41,55,89,90,91,92,93,94,95,96,97,98]. It is worth mentioning that apart from the last four formats, MIPs have to be immobilized on a transducer surface following the preparation. 

Over the years, classical polymerization techniques have been successfully applied for low-molecular-weight substances and even resulted in commercial products [99]. Nonetheless, commercial MIPs for routine analysis of biomacromolecules such as proteins, nucleic acids, bacteria, and viruses are still challenging, although successful examples have been presented in the literature [6,20,39,58,100,101,102,103,104,105]. This difficulty mainly arises from the stability problems faced during the imprinting process. As they are fragile and have a complex structure, polymerization can lead to structural changes including denaturation, unfolding, or aggregation. Another obstacle to the classical imprinting process is that the template molecules may be fully entrapped in the polymer matrix, thus hindering their removal and rebinding. For this purpose, milder imprinting techniques such as soft lithography and electropolymerization have been exploited. Both techniques have been utilized both for proteins and viruses [20,37,55,56,82,106,107,108,109,110,111,112].

Soft lithography, which has been introduced by Bain and Whitesides, is one of the elegant ways for the preparation of MIPs on the surface of transducers [39,113,114]. It allows the formation of micro- and nanopatterns. The key elements of soft lithography techniques are elastomeric stamps or mold, in which flexible organic materials are used rather than rigid inorganic materials [115]. Moreover, it is, in some manner, superior to photolithography due to its cost-effectiveness and easy adaption for patterning surfaces in the range from micro- to nanometers [55,66,114,115,116,117]. In the literature, various successful examples of this method have been presented for different viruses such as tobacco mosaic virus, subtypes of influenza a virus, picornavirus, dengue type 1 virus, and classical swine fever virus [37,56,109,118,119].

Electropolymerization is another elegant and widely applied technique for the preparation of MIP-based biomimetic sensors for both low-molecular-weight analytes and biomacromolecules and, to some extent, for viruses, as it allows the preparation of MIPs directly on a transducer’s surface under mild conditions [19,20,46,62,106,108,120,121,122,123,124,125,126,127]. The thickness of the polymeric film can be easily adjusted by just simply controlling the charge passed through, which results in more effective template removal and rebinding processes. Moreover, there is no need for cross-linkers.

(3) Template removal

The template removal step is as crucial as polymerization. Incomplete removal can result in reduced binding efficiency due to the smaller number of free binding cavities, while complete removal trials may cause partial or complete destruction of the polymeric network [20,128,129]. Unfortunately, there is no general removal procedure for MIPs such as that described for the regeneration of aptamers [20]. For decades, different strategies such as Soxhlet extraction, changing the pH or ionic strength, detergents, electrochemical methods, proteolytic digestion, elevated temperature, ultrasound, microwave-assisted extraction, and supercritical CO_2_ have been demonstrated. Extensive information about this topic can be found elsewhere [128,129].

## 3. MIP-Based Biomimetic Sensors for SARS-CoV-2 Detection

Basically, two different procedures have been described in the literature for the MIP-based biomimetic sensors against low-molecular-weight substances, biomacromolecules, and viruses. The first procedure is based on two steps of MIP preparation: (i) synthesis of MIPs separately and (ii) integration of the synthesized MIPs on a transducer [20].

Alternatively, diverse surface imprinting methods including the aforementioned methods such as soft lithography, electropolymerization, and self-polymerization have been developed, which allows direct preparation of the MIPs on the surface of the transducer. 

The integration of nanomaterials increases the active surface area and thereby enhances the sensitivity of the sensors. For this reason, a variety of nanomaterials, such as magnetic nanoparticles, carbon nanomaterials (carbon nanotubes, graphene, and its derivatives), metallic nanoparticles (Au nanoparticles (NPs), PtNPs, AgNPs), quantum dots, and nanocomposites, have been presented in the literature [130,131,132,133,134,135]. 

The recognition of analytes by MIPs has been coupled with a broad variety of transducers, among which electrochemical and optical transduction systems clearly dominate [19,20,104,136,137,138]. Nevertheless, for virus sensing, piezoelectric transducers find a wider application [136]. However, to the best of our knowledge, a QCM-based MIP sensor for the detection of SARS-CoV-2 has not been reported in the literature up to now. By contrast, electrochemical transducers dominate. In the following sections, we describe the MIP-based biomimetic sensors against SARS-CoV-2 considering the structural levels of the target analyte as described above (Table 1). 

### 3.1. Electrochemical Detection of SARS-CoV-2

Electrochemical approaches are easy to apply and, due to the smaller size of instruments, experiments can be performed without the need for professional personnel or well-equipped laboratories. 

Among the diverse approaches for the detection of analytes with MIP-based electrochemical sensors, voltammetric methods are widely utilized. By contrast, the number of potentiometric transducers, capacitors, or field-effect transistors is lower [19]. It should be noted that the potential window of voltammetric sensors is restricted by the cathodic hydrogen generation and the anodic oxygen evolution. Hence, the electrode material should be considered. 

Three main electrochemical readout methods have been utilized for MIP-based sensors [19]: (i) direct measurement of electroactive analytes (low-molecular-weight analytes, proteins); (ii) measurement of the signal generated by catalytically active analytes (enzymes); (iii) indirect measurement using a redox marker such as ferricyanide, ferrocene, and ruthenium (low-molecular-weight molecules, biomacromolecules, viruses, bacteria, cells), which is based on the gate effect [145,146]. This effect was, for the first time, described by Yoshimi et al. [145]. However, the mechanism is still under discussion [146]. Among the described readout methods, the last one is appropriate for virus sensing. Differential pulse voltammetry (DPV), square-wave voltammetry (SWV), or cyclic voltammetry (CV) play an important role in virus sensing. In comparison to CV, DPV and SWV are more sensitive as they allow the elimination of the charging current. In addition, electrochemical impedance spectroscopy (EIS) has been utilized in MIP-based virus sensing. 

(1) Whole virus imprinting: Despite the challenges of whole virus imprinting, some successful examples have been presented for SARS-CoV-2 sensing.

Hassan’s group proposed a MIP-based sensor against the whole SARS-CoV-2 particles [139]. The sensor was fabricated by electropolymerization of a mixture containing 3-aminophenol and virus particles on a carbon nanotube (CNT)/WO_3_-modified screen-printed carbon electrode. Steps of the MIP preparation were characterized by EIS in a solution of double redox mediators ferricyanide and DCIP. LOD and LOQ values were determined to be 57 and 175 pg/mL, respectively. Furthermore, almost no cross-reactivity was observed toward H1N1, H5N1, and H3N2 influenza A viruses, whereas MERS-CoV and the other human coronaviruses resulted in about 2 and 36% of cross-reactivity, respectively. Moreover, the virus-imprinted sensor can rapidly quantify the SARS-CoV-2 concentration in clinical samples and differentiate between the healthy and infected samples. By comparing the LODs, the authors claimed to obtain an almost 27-fold higher sensitivity compared to RT-PCR.

Recently, Reddy’s group presented an MIP-based sensor on an SPE, which was prepared by electropolymerization of N-hydroxmethylacrylamide (NHMA) against the whole SARS-CoV-2 virus [140]. By contrast, pseudoparticles and a cross-linker (N, N’-methylenebisacrylamide) have been utilized as a template and cross-linker, respectively. The sensor was characterized by EIS. The linear dynamic range was found to be log_10_ 4.0–6.0 pfu/mL with an LOD of 4.9 log10 pfu/mL. Furthermore, the developed sensor was exploited to real patient saliva samples. Positive and negative cases could be discriminated from the Nyquist plot and the results were an overall 75% agreement with the established loop-mediated isothermal nucleic acid amplification technique used by the UK National Health Service.

**Figure 4:**
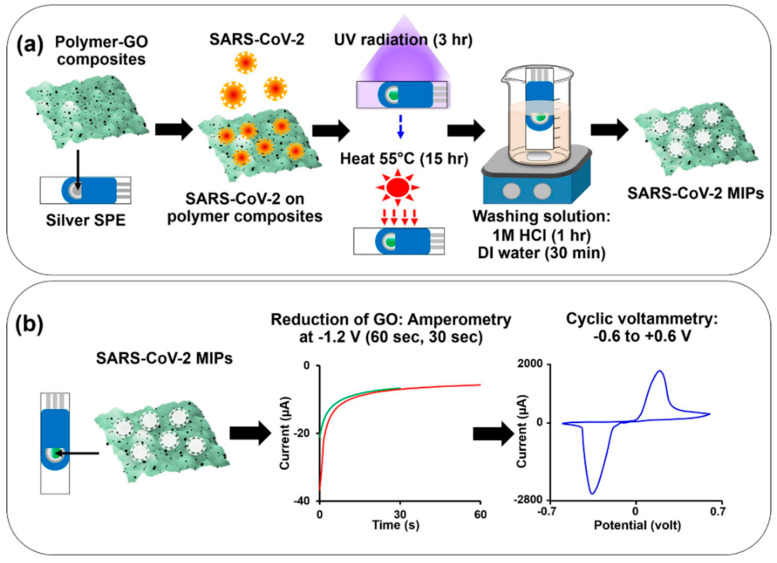
Schematic representation of (**a**) the preparation of MIP-based sensor and (**b**) the electrochemical reduction of graphene oxide Reprinted with permission from [141]. 2022, Elsevier.

In another work, inactivated SARS-CoV-2 was used as a template for the electrochemical detection of SARS-CoV-2 in water samples [141]. In comparison to the last two sensors, four different functional monomers and a cross-linker were used (Figure 4). An MIP-based sensor was prepared as follows: First, functional monomers and the cross-linker were mixed and heated. In the next step, graphene oxide was added to the mixture and dropped on SPE. In the last step, the template was dropped on the electrode, and polymerization was started by applying UV radiation. After template removal, graphene oxide was reduced electrochemically. A calibration curve was prepared based on the cyclic voltammetric response of the redox marker ferri-/ferrocyanide. The LOD was calculated to be 0.1 fM in buffer and wastewater spiked with SARS-CoV-2. Moreover, the sensor demonstrated, to some extent, a higher sensitivity to influenza A H5N1 virus.

(2) Functional viral protein (N- or S-Protein) imprinting: Raziq et al. described the first biomimetic sensor for the diagnosis of SARS-CoV-2 since the outbreak of COVID-19, which employed an electrochemical transducer. As a template, a functional viral protein, namely nucleoprotein (ncovNP), was used rather than the whole virus [63]. The MIP was prepared after covalent immobilization of ncovNP on a 4-aminothiophenol (4-ATP)-modified gold electrode. In the literature, it was described that the oriented immobilization of the target prior to polymerization via site-specific anchors allows the formation of more uniform binding cavities (grafted target imprinting) [20,147]. It can also prevent the denaturation of viral proteins due to the absorption on the metal surface. Moreover, thiol groups on the virus’ surface may lead to nonspecific interactions with a metal electrode. After molecular docking and quantum chemical calculations, m-phenylenediamine was chosen as the functional monomer. All the steps of MIP preparation were characterized with a redox marker, ferri-/ferrocyanide mixture. Limit of detection (LOD) and limit of quantification (LOQ) were calculated from DPVs to be 15 fM and 50 fM, respectively, which lies in the clinical range. Further, the selectivity studies with various proteins possessing different sizes, molecular weight, and isoelectric points demonstrated that the highest response was obtained for the target analyte ncovNP. Moreover, the performance of the sensor was exploited in nasopharyngeal swab specimens and a correlation with RT-PCR was found. 

Another electrochemical MIP-based sensor, which explored ncovNP as a template, was constructed on a gold/graphene nanohybrid-modified screen-printed electrode by electropolymerization of arginine. DPV was applied to characterize the sensor. Under the optimized conditions, the peak currents of the redox marker decreased with the increase in the ncovNP concentration from 10.0 and 200.0 fM with a very small LOD value of 3.0 fM, which was fivefold less than the previous example (15 fM). Furthermore, the sensor was applied to artificial nasal and saliva samples spiked with ncovNP, and detection of ncovNP was achieved with acceptable recovery values.

In addition to the N protein of SARS-CoV-2, the S protein was successfully applied as a template for the construction of an electrochemical biomimetic sensor by Ramanavicius’s group. For the fabrication of the sensor, they utilized pyrrole, which forms conductive polymeric films by electropolymerization on a Pt electrode [62]. After removal of the template molecules with sulfuric acid, the performance of the sensor was evaluated by means of amperometry applying pulse values of 0 V and +0.6 V. A linear response was observed upon rebinding of the template S protein of SARS-CoV-2 ranging from 0 μg/mL to 25 μg/mL. The imprinting factor was determined to be approximately 2.1. Moreover, the developed MIP showed a significantly higher sensitivity toward its template as compared to bovine serum albumin. 

**Figure 5:**
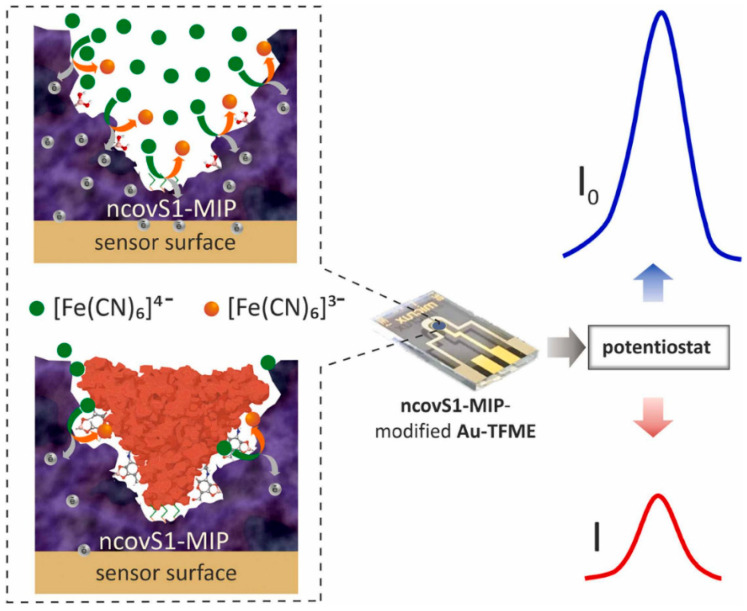
Representation of the measurement in a redox marker solution upon the rebinding of the target analyte. Reprinted with permission from [65]. 2022, Elsevier.

(i) Subunit of the S protein imprinting: The use of N protein as a target may cause false-positive results as shown elsewhere [148]. Therefore, despite the success of the aforementioned method [63], Syritski’s group developed an MIP-based sensor addressing the spike protein subunit S1 (Figure 5) [65]. Prior to polymerization, the template was immobilized on a modified electrode. Moreover, the authors took advantage of the covalent interaction between 1,2-diols of the highly glycosylated protein and the boronic acid by using 3-aminophenylboronic acid (APBA) as a functional monomer. The sensor was evaluated by the concentration-dependent current suppression of redox marker ferri/ferrocyanide via SWV (Figure 5). The sensor had a quick response time of 15 min detecting ncovS1 in buffer and also in nasopharyngeal samples in fM levels (Table 1).

In another study, Tabrizi et al. suggested an ultrasensitive MIP-based electrochemical sensor for the detection of the receptor-binding domain (RBD) of SARS-CoV-2 [64]. The MIP was constructed by electropolymerization on a microporous gold screen-printed electrode (MP-Au-SPE). RBD and o-phenylenediamine were template and functional monomers, respectively. Each step of the imprinting process was analyzed using EIS and CV. The sensor showed a linear response from the 2.0 pg·mL^−1^ level to 40 pg·mL^−1^ with an LOD value of 0.7 pg·mL^−1^. The authors further applied the MIP sensor to the saliva sample and compared it with ELISA and found no significant difference between the two methods. 

### 3.2. Optical Detection of SARS-CoV-2

In addition to the electrochemical readout, the optical readout was exploited for the detection of SARS-CoV-2 with MIP-based sensors. Optical sensors allow the direct detection (label-free) of analytes by measuring the changes such as refractive index and fluorescence. Compared to electrochemical MIP-based sensors, the number of optical MIP-based sensors against SARS-CoV-2 is limited. 

Cennamo et al. reported for the first time an acrylamide-based MIP on a POF-covered gold SPR chip addressing the specific recognition of the S1 subunit of the SARS-CoV-2 spike protein [144]. This first prototype was exploited to detect the S1 subunit. The LOD and affinity constants were determined to be 0.058 µM and 2.318 µM^−1^, respectively. Moreover, preliminary tests on SARS-CoV-2 virions were performed on samples of nasopharyngeal swabs in the universal transport medium and physiological solution (0.9% NaCl), and the results were compared with RT-PCR. They obtained a higher sensitivity and faster response. However, the authors expressed that the method should be validated.

**Figure 6:**
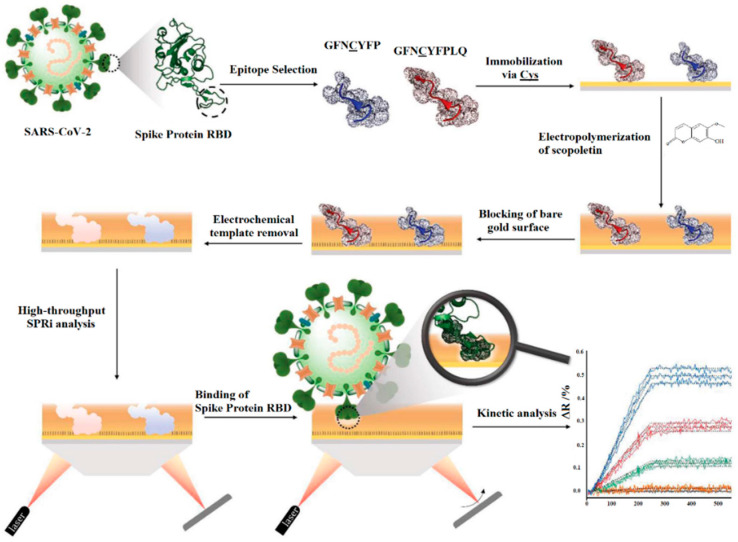
Schematic representation of steps of virus imprinting procedure preparation and the SPR responses of the rebinding Reprinted with permission from [82]. 2022, RSC.

In another study, Bognar et al. developed an MIP sensor, which applied the nonapeptide 485–493 of the RBD as the epitope template (Figure 6) [82]. The peptide GFNCYFPLQ was microspotted on gold SPR chips before the deposition of polyscopoletin. Template removal was achieved by anodic potential pulses. The parent protein RBD was bound in the lower nanomolar concentration range, while the same concentrations of human serum albumin (HSA) had no effect in the 0.05 % Twin-20 solution. MIPs prepared with a peptide without the C-terminal L and Q showed a moderately decreased affinity. Substitution of the central C by S in the template peptide resulted in MIPs with no binding of both the RBD and HSA. Obviously, the central C is essential for the formation of “open” cavities that accommodate the 26 kDa protein. Furthermore, the RBD was indicated in the spiked splitting solution.

### 3.3. Commercial MIP for SARS-CoV-2

Several successful examples of nanoMIPs have been exploited in bioanalysis for the development of optical and electrochemical sensors [137,149,150]. The company MIP Diagnostics has developed the first commercial nanoMIPs against different SARS-CoV-2 variants, addressing the RBD of SARS-CoV-2. The particle size varies from 40 nm to 80 nm and the affinity constants are ≤18. As the nanoMIPs are amino-functionalized, they could be immobilized on an electrode. The developed thermal resistance sensor allowed the measuring of concentrations of <5 fg/mL for the RBD from spike protein. 

## 4. Conclusions

MIPs have received growing attention over the past decades for the substitution of biological reagents in separation techniques, bioanalysis, and biotechnology to overcome limitations faced in analysis. They are easy to prepare, cost-effective, and stable under harsh conditions. Moreover, in contrast to antibody production, animals are not required. Taking into account the advantages, several MIP-based biomimetic sensors against SARS-CoV-2 have been described in the literature, and even the company MIP Diagnostics produced commercial nanoMIPs. In this review, we summarized these biomimetic sensors and grouped them according to readout methods. In addition, grouping structural levels of the target analyte from the whole virus to its epitope was also taken into consideration. It is worth mentioning that utilizing an epitope as a template, on the one hand, can provide some advantages but, on the other hand, may result in nonspecific interaction of the whole virus with the polymer. RT-PCR is so far the gold-standard method for the determination of the viral load of SARS-CoV-2, and it is still under question whether the described MIPs can replace it. On the other hand, combining MIPs with lateral flow assays can be realized as a cheaper and more stable alternative to antigen tests. 

## Figures and Tables

**Table 1:** MIP-based biomimetic sensors for SARS-CoV-2 detection.

Template	Monomer	Transducer	Detection Method	(Linear) Range and LOD	Ref.
SARS-CoV-2 whole virus	3-AP	CNT/WO_3_-SPCE	EIS	LOD: 57 pg/mL	[139]
SARS-CoV-2 whole virus	NHMA MBAm (cross-linker)	SPE	EIS	3–7 log_10_ pfu/mL LOD: 4.9 log_10_ pfu/mL	[140]
SARS-CoV-2 whole virus	AAM, MAA, MMA, and NVP; DHEBA (cross-linker)	GO integrated Ag-SPE	CV	0.01 fM to 100 fM LOD: 0.1 fM	[141]
SARS-CoV-2 whole virus	Pyrrole; (graphene oxide) APBA (cross-linker);	GCE	DPV and amperometry	DPV: 0.74–9.03 fg mL^–1^ and LOD: 0.326 fg mL^–1^ Amperometry: 13.14–118.9 fg mL^–1^ and LOD: 11.32 fg mL^–1^	[142]
SARS-CoV-2 nucleoprotein	m-PD	4-ATP-modified Au-TFE	DPV	Up to 111 fM; LOD: 15 fM (in lysis buffer)	[63]
SARS-CoV-2 nucleocapsid protein	Arginine	Au/Gr-modified SPCE	DPV	10.0–200.0 fM; LOD: 3 fM	[143]
SARS-CoV-2 spike protein	Pyrrole	Pt Electrode	CA	0 μg/mL to 25 μg/mL	[62]
SARS-CoV-2 RBD	o-PD	MP-Au-SPE	EIS	2.0 pg·mL^−1^–40 pg·mL^−1^ LOD: 0.7 pg·mL^−1^	[64]
SARS-CoV-2 spike protein subunit S1	APBA	4-ATP-modified Au-TFME	SWV	LOD: 15 fM (in PBS) and 64 fM (patient’s nasopharyngeal samples)	[65]
SARS-CoV-2 spike protein subunit S1	Aam, TBAm, and HEMA; BIS (cross-Linker)	POF-based SPR chip	SPR	LOD: 0.058 µM	[144]
SARS-CoV-2 spike protein RBD epitope (GFNCYFPLQ)	Scopoletin	Au- SPRi chips	SPR	NS	[82]

3-AP: 3-aminophenol; AAM: Acrylamide; APBA: 3-aminophenyl- boronic acid; Au-TFME: Thin-film Au metal electrodes, BIS: N,N′-methylene bisacrylamide; CA: Chronoamperometry; DHEBA: N,N′-(1,2-dihydroxy- ethylene) bisacrylamide; HEMA: 2-hydroxyethyl methacrylate; LOD: Limit of Detection; LOQ: Limit of Quantification; MAA: Methacrylic acid; MBAm: N,N′-methylenebisacrylamide; MMA: methyl methacrylate; m-PD: m-Phenylenediamine; NHMA: N-hydroxmethylacrylamide; NS: Not stated; NVP: N-vinylpyrrolidone; o-PD: o-Phenylenediamine; POF: Plasmonic Optical Fibers; SPCE: screen-printed carbon electrode; TBAm: N-t-butylacrylamide.

## Data Availability

Not applicable.
